# The association between triglyceride glucose index and depression: data from NHANES 2005–2018

**DOI:** 10.1186/s12888-021-03275-2

**Published:** 2021-05-25

**Authors:** Yi-Yi Shi, Rui Zheng, Jie-Jie Cai, Song-Zan Qian

**Affiliations:** 1 Department of Anesthesiology, The First Affiliated Hospital of Wenzhou Medical University, Wenzhou, 325000 China; 2 Department of Intensive Care Unit, The First Affiliated Hospital of Wenzhou Medical University, Wenzhou, 325000 China; 3 Wenzhou Key Laboratory of Critical Care and Artificial Intelligence, Wenzhou, China

**Keywords:** Depression, Depressive symptoms, Triglyceride glucose index, Insulin resistance, NHANES

## Abstract

**Background:**

The association between triglyceride glucose (TyG) index and depression is unclear. We conducted this analysis to explore whether higher TyG index is associated with a higher odd of depression.

**Methods:**

This was an observational study using data from the National Health and Nutrition Examination Survey (2005–2018), a cross-sectional and nationally representative database. Depression was assessed using the Patient Health Questionnaire-9 (PHQ-9). TyG index was calculated based on the equation as follows: ln [triglyceride (mg/dL) × fasting blood glucose (mg/dL)/2], and participants were divided into quartiles based on TyG index. Weighted multivariable logistic regression models were used to explore the relationship between the TyG index and depression.

**Results:**

A total of 13,350 patients were included, involving 1001 (7.50%) individuals with depression. Higher TyG index is significantly associated with elevated depressive symptoms in U.S. adults. Multivariate-adjusted HRs for patients in the TyG index 4th quartile were higher for depression (OR = 1.46; 95% confidence interval (CI) 1.30, 1.64) compared with the 1st quartile of TyG index. Similar results were seen in men and women, across age groups, and baseline comorbidities.

**Conclusion:**

In this large cross-sectional study, our result suggests that population with higher TyG index are significantly more likely to have depressive symptoms in U.S. adults.

**Supplementary Information:**

The online version contains supplementary material available at 10.1186/s12888-021-03275-2.

## Introduction

Depression is a common mental health issue, clinically characterized by significant and persistent low mood symptoms, linked with considerably diminished role-functioning and quality of life, and with high risk of medical comorbidity and mortality [1]. Nearly 17.3 million adults had experienced at least one major depression in the USA in 2017. The prevalence was about 7.1%, and highest among adults reporting two or more races [2]. According to the Depression and Other Common Mental Disorders: Global Health Estimates published by WHO in 2017, there were 322 million people living with depression in the world. Meanwhile, the prevalence of depression was estimated above 7.5% among females aged 55–74 years [3]. A study on the incidence of depression in the United States showed a high prevalence of depression, and misperception of depression existing among the US adult population [4]. A substantial number of researches have shown strong relationships between depression and physical health, including Parkinson’s disease, metabolic disease, diabetes, dementia and cancer [3, 5–9].

Insulin resistance is an obvious feature of type 2 diabetes, hypertension, lipid metabolism disorders and even cardiovascular disease, which means decreased responsiveness to insulin. Some large population of observational studies found that diabetes was related to depression caused by insulin resistance [10–12]. The triglyceride-glucose (TyG) index is a new indicator of insulin resistance in recent years. Studies show that TyG index is related to disease prognosis, cardiovascular disease, dementia [12, 13]. However, the association between triglyceride glucose (TyG) index and depression is still unclear. Therefore, the purpose of this study is to explore the relationship between TyG index and depression in a large, nationally-representative sample of adults living in the United States. The dataset from the National Health and Nutrition Examination Survey (NHANES) was collected from 2005 to 2018.

## Material and methods

### Subjects and study design

**Fig. 1:**
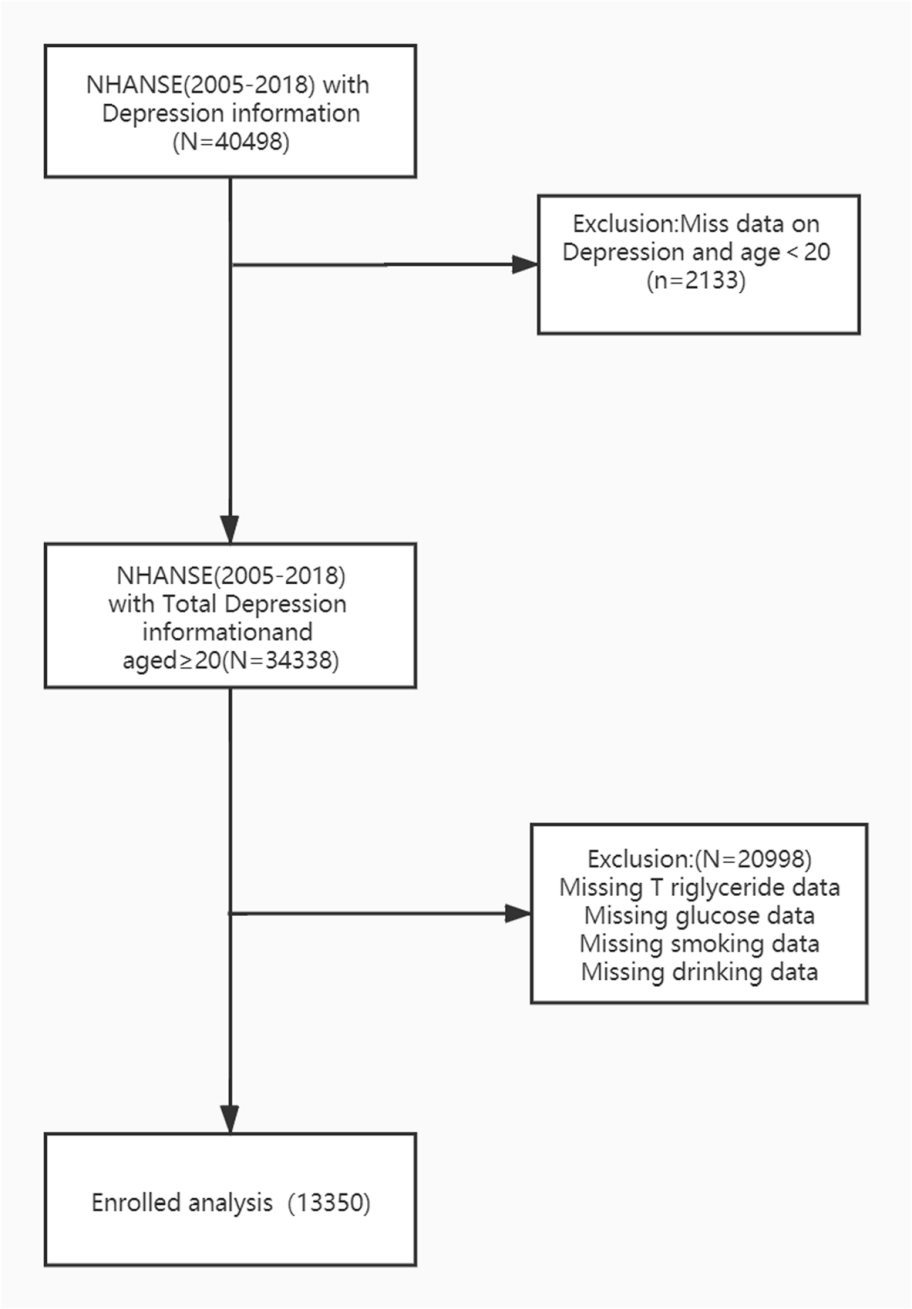
Flow chart of subject selection

This is a cross-sectional study, the data comes from the National Health and Nutrition Examination Survey (2005–2018). The examination components consist of medical, dental, and physiological measurements, and laboratory tests supervised by trained medical personnel. Furthermore, the adoption of various modern equipment enables the NHANES to collect reliable, high-quality data. Moreover, compensation and a report of medical findings are given to each participant, which increases the compliance of participants [2]. The total sample size of adults from the 2005–2018 assessments is 13,350. Additional details of the study design, sampling, and exclusion criteria are described in Fig. 1. Only publicly available data is used in the analysis, and the protocol of NHANES is approved by the National Center for Health Statistics (NCHS) Research Ethics Review Board.

### Data collection and definitions

Based on the existing literature [2, 14], variables that might potentially confound between TyG index and depression symptoms are assessed: sex, age, race/ethnicity, education level. The Poverty Income Ratio (PIR) is used as a proxy measure for socioeconomic status (SES). Smoking status is categorized into never smoker (reference group), former smoker, and current smoker. Drinking status is divided into none (reference group), light, moderate, and heavy alcohol consumption, respectively according to the daily drinking volume (0, < 1, 1–< 8, and > 8 drinks per week). Body Mass Index (BMI) was divided to three groups: normal < 25.0, overweight 25.0–29.9, obese 30.0+. Congestive heart failure, coronary heart disease, cancer or malignancy, liver condition diabetes (Doctor informed you had any condition yes vs no).

TyG index is calculated by the following formula: TyG = Ln [fasting triglycerides (mg/dL) × fasting glucose (mg/dL)/2] [13]. The depressive state was assessed using the Patient Health Questionnaire (PHQ-9) in NHANES and When PHQ-9 score ≥ 10, the interviewee is considered to have depression.

### Statistical analysis

The presentation of continuous variables was divided according to their distribution status. Weighted means (standard error) were calculated for continuous variables, and weighted frequency percentages were calculated for categorical variables [15]. Chi-squared test or Kruskal-Wallis H test was employed to different TyG index quartile groups. Any non-linear relationships between TyG index levels and depression were assessed using restricted cubic spline curves. The association between TyG index levels and depression was evaluated by logistic analysis. Logistic regression models were used to calculate the odds ratio (OR) to estimate the association between TyG index levels and depression.

These covariates were chosen according to their relationship with the outcomes of interest or > 10% effect estimate changes [16]. We constructed three models; Non-adjusted model; Model 1 adjust for: Age (years); Sex; Race; Model 2 adjust for: Age (years); Race; Sex; Education level; Household income; BMI status; Drinking status; Smoking status; congestive heart failure; coronary heart disease; liver condition; cancer or malignancy; Diabetes; HDL cholesterol (mg/dL).

Stratified analyses by subgroup variables were presented with a fully adjusted Model 2. Log-likelihood ratio test was used to assess the interaction effects between TyG index and subgroup variables.

Weighted analyses were applied to account for the complex sampling design of NHANES. The detail was in this website as followed: https://wwwn.cdc.gov/nchs/nhanes/tutorials/module3.aspx

All analyses were performed with the statistical software EmpowerStats (http://www.empowerstats.net/cn/index.php) and R version 3.6.1 (https://www.r-project.org/). a two-side *p*-value of < 0.05 was considered statistically significant in all analyses.

## Results

### Baseline characteristics of study participants based on the TyG index

Figure 1 described the study design, sampling, and exclusion; and 23,131 participants were excluded because of missing data on any of the covariates (missing data on Depression; TyG index, smoking status, drinking status). Our final sample included 13,350 NHANES participants, with 48.50% females and 70.50% non-Hispanic White. There were 1001 (7.50%) individuals with depression. Baseline characteristics of study participants based on the TyG index quartiles are shown in Table 1. Among all participants, the TyG index across quartiles was positively associated with age, BMI, current smoking, alcohol consumption, household income high prevalence of diabetes, congestive heart failure, coronary heart disease, liver condition, cancer or malignancy (all *p* < 0.001). In addition, regular HDL cholesterol was negatively associated with the TyG index across quartiles (all *p* < 0.001).

**Table 1:** Weighted baseline characteristics according to triglyceride-glucose index quartile

TyG index quartile	Total	Q1	Q2	Q3	Q4
N	13,350	3097	3317	3451	3485
**Age (years) group**					
< 60	74.20	82.40	74.20	71.20	69.40
> =60	25.80	17.60	25.80	28.80	30.60
**Sex**					
Male	51.50	40.70	49.80	54.50	60.50
Female	48.50	59.30	50.20	45.50	39.50
**Race**					
Mexican American	8.10	5.90	7.10	8.90	10.30
Other Hispanic	5.30	5.20	5.20	5.40	5.50
Non-Hispanic White	70.50	66.40	720	71.20	72.10
Non-Hispanic Black	9.90	15.80	10.40	7.30	6.10
Other Race	6.30	6.70	5.20	7.20	60
**Education level**					
Less than 9th grade	4.40	2.70	3.70	4.80	6.50
9-11th grade	10.30	7.50	10.20	11.30	12.10
High school graduate/GED or equivalent	23.20	19.50	22.90	24.30	26.0
Some college or AA degree	31.80	32.30	30.80	310	32.90
College graduate or above	30.30	37.90	32.30	28.60	22.60
**Household income**					
0–130% FPL	19.50	18.50	190	19.90	20.80
> 130–350% FPL	35.80	34.10	34.90	37.10	37.10
> 350% FPL	44.70	47.40	46.10	430	42.10
**BMI status**					
Normal or low weight	300	51.90	34.50	22.60	11.60
Overweight	33.30	280	36.20	34.70	34.20
Obese	36.60	20.10	29.30	42.60	54.30
**Smoking status**					
Never	50.10	58.90	51.60	48.10	420
Former	28.10	23.40	26.40	29.10	33.40
Current	21.80	17.70	21.90	22.80	24.60
**Drinking status**					
None	17.50	12.20	16.40	190	22.10
Light	44.40	47.30	43.50	44.20	42.40
Moderate	24.40	28.70	25.40	22.60	210
Heavy	13.80	11.80	14.70	14.10	14.50
**Triglyceride (mg/dL)**	126.4 (1.4)	53.9 (0.4)	86.9 (0.3)	126.7 (0.6)	242.8 (3.3)
**HDL cholesterol (mg/dL)**	54.3 (0.2)	64.6 (0.5)	57.5 (0.4)	51.4 (0.3)	43.7 (0.3)
**Glucose (mg/dL)**	106.0 (0.4)	94.5 (0.3)	99.9 (0.3)	104.9 (0.4)	125.7 (1.0)
**Diabetes**					
Yes	9.20	2.10	4.30	8.20	22.20
No	88.60	96.90	94.10	88.60	74.70
Borderline	2.20	10	1.60	3.20	3.20
**coronary heart disease**					
Yes	3.60	20	2.90	4.30	5.30
No	96.40	80	97.10	95.70	94.70
**liver condition**					
Yes	4.10	2.70	3.30	4.20	6.30
No	95.90	97.30	96.70	95.80	93.70
**congestive heart failure**					
Yes	2.50	1.40	20	2.50	40
No	97.50	98.60	980	97.50	960
**cancer or malignancy**					
Yes	9.90	7.60	10.60	9.50	11.80
No	90.10	92.40	89.40	90.50	88.20
**Depression**					
No	92.50	93.90	93.60	92.80	89.70
Yes	7.50	6.10	6.40	7.20	10.30

Values are means ± SE or percentages. All estimates were weighted to be nationally representative

### Associations between TyG index and depression

**Fig. 2:**
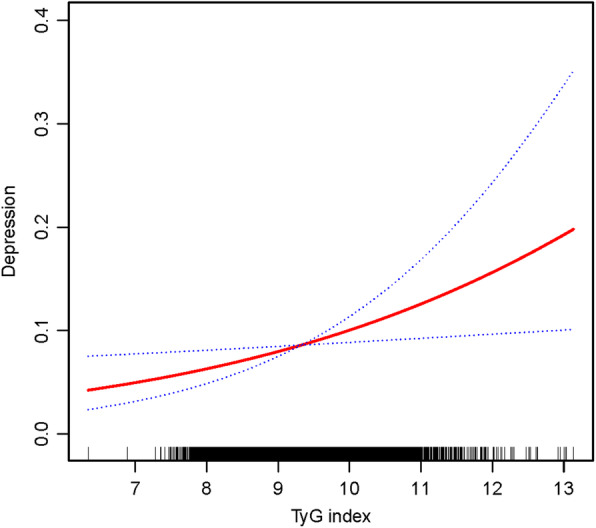
Restricted cubic spline fitting for the association between TyG index levels with depression

As shown in Fig. 2, Generalized Additive Models suggested that the relationship between TyG index and depression was linear. In Table 2, after adjusting for demographic characteristics, higher TyG index was associated with higher relative risks of depression. Compared to the 1st quartile of TyG index, multivariate-adjusted OR for patients in the TyG index 4th quartile had higher odds of having depression (OR = 1.46; 95% confidence interval (CI) 1.30, 1.64). Adults with diabetes, congestive heart failure, coronary heart disease, liver condition, cancer or malignancy were more likely to suffer from depression.

**Table 2:** Weighted relationship between Triglyceride-glucose index and depression

Exposure	Non-adjusted	Adjust I	Adjust II
TyG index	1.40 (1.33, 1.46) < 0.0001	1.55 (1.48, 1.62) < 0.0001	1.28 (1.20, 1.36) < 0.0001
TyG index quartile			
Q1	1.0	1.0	1.0
Q2	1.05 (0.95, 1.15) 0.3485	1.15 (1.05, 1.27) 0.0041	1.00 (0.90, 1.11) 0.9475
Q3	1.19 (1.08, 1.30) 0.0004	1.38 (1.25, 1.52) < 0.0001	1.07 (0.96, 1.19) 0.2511
Q4	1.75 (1.60, 1.91) < 0.0001	2.14 (1.95, 2.35) < 0.0001	1.46 (1.30, 1.64) < 0.0001
P for trend	< 0.0001	< 0.0001	< 0.0001

Data are presented as odds ratios, 95% confidence intervals, and *P*-value

Non-adjusted model adjusts for: None

Adjust I model adjust for: Age (years); Sex; Race

Adjust II model adjust for: Age (years); Race; Sex; Education level; Household income; BMI status; Drinking status; Smoking status; congestive heart failure; coronary heart disease; liver condition; cancer or malignancy; Diabetes; HDL cholesterol (mg/dL)

**Fig. 3:**
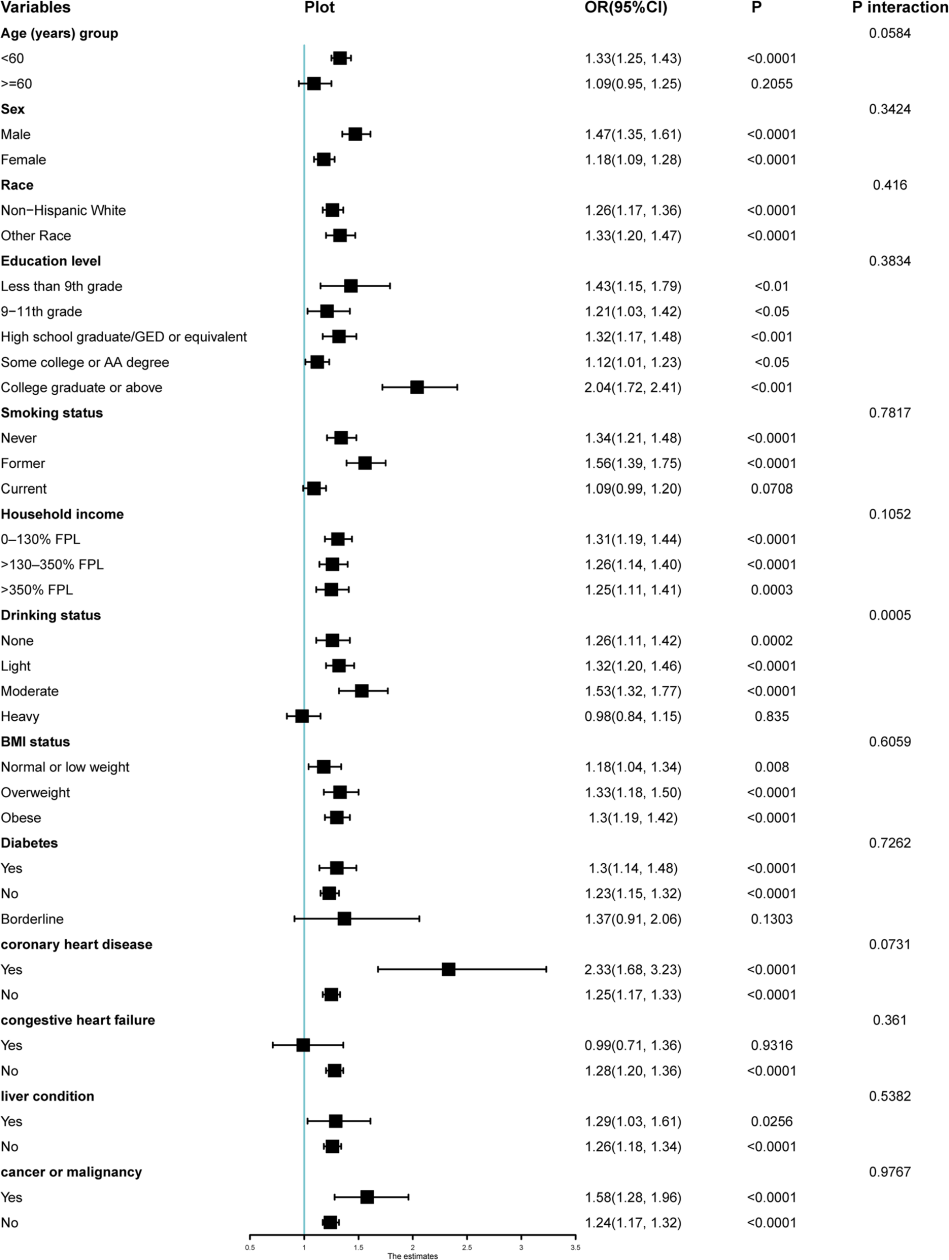
Odds of depression subgroups based on the increasing TyG index with various clinical variables

Subgroup analyses was performed to evaluate the robustness of the association between TyG index and depression. In the adjusted model 2, the results of stratified analysis showed that the association of TyG index was similar in the most of the sub-populations (Details in Fig. 3 and Table S1). The results of univariate analysis of depression were shown in Table S2, and the relationship between Triglyceride, glucose and depression was shown in Table S3.

## Discussion

In this national representative cross-sectional study, the association between TyG index and depression was assessed. The main finding of this article is that a high level of TyG was associated with a higher odds of having depression in the US adult population. The result is still consistent after different stratification analysis and sensitivity analysis. To our best knowledge, this is the first study to explore the association between the TyG index and depression in the general population.

The details of the mechanism explaining the relationship between the TyG index and depression still need to be further explored, and there may be several possible explanations as follows. Firstly, patients with depression have adverse insulin resistance (IR). A cross-sectional study involving more than 160,000 people in South Korea showed that higher risk of depression was associated with insulin resistance. As insulin resistance increases, the risk of depression in young adults and non-diabetic individuals increases by 4 and 17% respectively [17]. TyG index is another new indicator used to evaluate insulin resistance status in recent years. This present study was the first in which a positive association was observed between TyG index quartile and depression in a large population. The results of this study are similar to those previously published, based on HOMAIR index, an indicator of insulin resistance, in a large population. Meanwhile, the relationship between TyG and the depression is still consistent even in various subgroup analysis.

Secondly, Reverse causality may also explain the relationship between high level of TyG and higher odds of depression. Higher TyG index not only indicates insulin resistance, but also indicates adverse health conditions, associated with cerebrovascular disease, cardiovascular disease, obesity, diabetes, hypertension, metabolic syndrome, lipid metabolism disorders [18–20]. The disease state may also cause an increase in levels of triglycerides and blood sugar. The results of this study show that comorbidities are more frequent in individuals with the high levels of TyG index. Also, high levels of triglyceride are an indirect marker of severe disease. The association between high levels of TyG index and higher odds of having depression is the strongest in age, sex and race adjusted model, and is reduced when it is adjusted for baseline comorbidities. After excluding individuals known diabetes, congestive heart failure, coronary heart disease, liver condition, cancer or malignancy at baseline, the association remains after this adjustment.

Thirdly, inflammatory and oxidative stress may play an important role in the association of TyG index with depression. Previous observational studies have shown positive associations of TyG index with inflammatory indicators of white blood cells and C-reactive protein, and high TyG index is related to endothelial dysfunction, inflammation response, and oxidative stress [21]. Inflammation can damage vascular endothelium, meanwhile, oxidative stress also causes vascular endothelial damage, involving the occurrence and progression of a variety of diseases, such as vascular depression and dementia [22].

Our results play a very important role in further understanding the relationship between insulin resistance and depression, especially in the general population. Patient with insulin resistance are generally accompanied by disorders of blood glucose or lipid metabolism [21]. Blood lipids further induce insulin resistance. The possible explanation is glucose fatty acid cycle hypothesis [23, 24]. When compared to healthy controls, depressive individuals are found with higher triglycerides and lower HDL-cholesterol levels. A study by Jae-Hon Lee et al. found that triglycerides were likely causal risk factors for depression. The odds of being depression increased by 18% for per standard deviation increase in genetically-predicted triglycerides. Therefore, the occurrence of depression can be reduced by controlling triglyceride levels [25]. Compared with the HOMA-IR indicator, TyG index is a better indicator of arterial stiffness. TyG index can serve as a practical alternative of IR measurement [26]. Previous evidence indicates that depression has a certain correlation with dementia and Alzheimer’s disease (AD), and clarifies the risk factors of depression which will help to further reduce dementia and AD [27]. A large cohort study found that TyG index is a risk factor for dementia, independent of traditional cardiovascular risk factors [14]. TyG is both related to depression and dementia, and we need to further explore the common mechanism between them to help block the cross-influence in depression and dementia. Greater knowledge of the physiologic may open avenues for specific therapies of AD.

In this study, we did a comparison between those who had been included vs those who not (Detail in table s4 and s5). The results showed that there was no difference between the two groups only in “depression, drinking status, cancer or malignancy, congestive heart failure, and coronary heart disease”. We admitted that it might have selection bias, but we established three models by adjusting different variables. The direction of the models is consistent. Although there are certain flaws in sample selection, our conclusions are still reliable. This study provides a basis for future multi-center cohort studies on depression and TyG.

The present study equally experiences several limitations. Firstly, due to the cross-sectional nature, this study does not imply causality. The finding may not be directly extrapolated to ethnic groups and other regions. The result is mainly applicable in the United States. Secondly, only one-time participants self-report whether they have experienced depressive symptoms using the PHQ-9 at least for 2 weeks in the previous year. Moreover, depression symptoms include mild and severe, which may differ in their relations with TyG index. Furthermore, data on medications is not included which might affect depression as well as influence on lipid and glucose metabolism disorders. Finally, the design of the study is observational, and we cannot draw conclusions about the question of causality.

## Conclusions

High TyG index is associated with a higher odds of having depression in individuals in the general population, after adjustment for case complexity. This finding indicated that the TyG index may be an independent but minor predictor of depression development.

## Supplementary Information

**Additional file 1: Table S1.** Weighted stratification analysis of Triglyceride-glucose index with depression. **Table S2.** Weighted univariate analysis of depression. **Table S3.** Relationship between Triglyceride, glucose and depression. **Table S4.** Baseline characteristics between Exclude and Include individual. **Table S5.** Missing of variables.

## Data Availability

Raw data supporting the obtained results are available at the corresponding author.
