# Supplementary material for: The association between triglyceride glucose index and depression: data from NHANES 2005–2018
Source: BMC Psychiatry. 2021 May 25;21:267. doi: 10.1186/s12888-021-03275-2 (PMC8146990; doi:10.1186/s12888-021-03275-2)
Supplement: Supplementary file 1 — Additional file 1: Table S1. Weighted stratification analysis of Triglyceride-glucose index with depression. Table S2. Weighted univariate analysis of depression. Table S3. Relationship between Triglyceride, glucose and depression. Table S4. Baseline characteristics between Exclude and Include individual. Table S5. Missing of variables. [file 12888_2021_3275_MOESM1_ESM.docx]

**Supplementary tables**

**Content:**

**Table S1.** Weighted stratification analysis of Triglyceride-glucose index with depression.

**Table S2.** Weighted univariate analysis of depression

**Table S3.** Relationship between Triglyceride, glucose and depression.

**Table S4.** Baseline characteristics between Exclude and Include individual

**Table S5.** Missing of variables

**Table S1.** Stratification analysis of Triglyceride-glucose index with depression.

| X= TyG index |  |  |  |
| --- | --- | --- | --- |
|  | N | DEPRESSION | P interaction |
| Age (years) group |  |  | 0.8658 |
| <60 | 8940 | 1.40 (1.34, 1.48) <0.0001 |  |
| >=60 | 4410 | 1.42 (1.29, 1.57) <0.0001 |  |
| Sex |  |  | 0.0190 * |
| Male | 7058 | 1.34 (1.25, 1.44) <0.0001 |  |
| Femal | 6292 | 1.63 (1.53, 1.73) <0.0001 |  |
| Race |  |  | 0.4160 |
| Mexican American | 2046 | 1.47 (1.26, 1.72) <0.0001 |  |
| Other Hispanic | 1292 | 1.57 (1.34, 1.83) <0.0001 |  |
| Non-Hispanic White | 6100 | 1.44 (1.36, 1.53) <0.0001 |  |
| Non-Hispanic Black | 2675 | 1.29 (1.14, 1.46) <0.0001 |  |
| Other Race | 1237 | 1.41 (1.17, 1.68) 0.0002 |  |
| Education level |  |  | 0.3834 |
| Less than 9th grade | 1172 | 1.27 (1.09, 1.48) 0.0021 |  |
| 9-11th grade | 1896 | 1.11 (0.98, 1.25) 0.1057 |  |
| High school graduate/GED or equivalent | 3092 | 1.30 (1.19, 1.43) <0.0001 |  |
| Some college or AA degree | 3996 | 1.23 (1.14, 1.32) <0.0001 |  |
| College graduate or above | 3184 | 1.78 (1.57, 2.01) <0.0001 |  |
| Household income |  |  | 0.7817 |
| 0–130% FPL | 3635 | 1.35 (1.26, 1.46) <0.0001 |  |
| >130–350% FPL | 4696 | 1.35 (1.25, 1.46) <0.0001 |  |
| >350% FPL | 3966 | 1.45 (1.32, 1.59) <0.0001 |  |
| BMI status |  |  | 0.1052 |
| Normal or low weight | 3830 | 1.34 (1.21, 1.48) <0.0001 |  |
| Overweight | 4441 | 1.35 (1.22, 1.48) <0.0001 |  |
| Obese | 4968 | 1.35 (1.27, 1.45) <0.0001 |  |
| Smoking status |  |  | 0.0232 * |
| Never | 6581 | 1.44 (1.34, 1.56) <0.0001 |  |
| Former | 3738 | 1.66 (1.52, 1.81) <0.0001 |  |
| Current | 3031 | 1.07 (0.99, 1.16) 0.0678 |  |
| Drinking status |  |  | 0.6059 |
| None | 2930 | 1.38 (1.26, 1.50) <0.0001 |  |
| Light | 6012 | 1.38 (1.29, 1.48) <0.0001 |  |
| Moderate | 2797 | 1.55 (1.40, 1.72) <0.0001 |  |
| Heavy | 1611 | 1.04 (0.92, 1.18) 0.5173 |  |
| Triglyceride (mg/dL) group |  |  | 0.7262 |
| 10 - 81 | 4434 | 0.92 (0.76, 1.10) 0.3588 |  |
| 82 - 131 | 4429 | 1.30 (1.02, 1.65) 0.0346 |  |
| 132 - 2742 | 4487 | 1.38 (1.26, 1.51) <0.0001 |  |
| HDL cholesterol (mg/dL) group |  |  | 0.0731 |
| 6 - 44 | 4150 | 1.45 (1.35, 1.57) <0.0001 |  |
| 45 - 57 | 4512 | 1.32 (1.21, 1.45) <0.0001 |  |
| 58 - 226 | 4688 | 1.38 (1.24, 1.53) <0.0001 |  |
| Glucose (mg/dL) group |  |  | 0.3610 |
| 21 - 95 | 4256 | 1.39 (1.26, 1.53) <0.0001 |  |
| 96 - 106 | 4618 | 1.25 (1.14, 1.37) <0.0001 |  |
| 107 - 584 | 4476 | 1.50 (1.40, 1.61) <0.0001 |  |
| Diabetes |  |  | 0.5382 |
| Yes | 1687 | 1.49 (1.35, 1.65) <0.0001 |  |
| no | 11332 | 1.26 (1.19, 1.33) <0.0001 |  |
| Borderline | 321 | 1.09 (0.83, 1.42) 0.5369 |  |
| coronary heart disease |  |  | 0.9767 |
| Yes | 558 | 1.08 (0.90, 1.31) 0.3999 |  |
| No | 12743 | 1.41 (1.34, 1.47) <0.0001 |  |
| liver condition |  |  | 0.2932 |
| Yes | 588 | 1.01 (0.87, 1.17) 0.9445 |  |
| No | 12731 | 1.41 (1.34, 1.48) <0.0001 |  |
| congestive heart failure |  |  |  |
| Yes | 438 | 1.01 (0.84, 1.22) 0.8871 |  |
| No | 12884 | 1.41 (1.35, 1.48) <0.0001 |  |
|  |  |  |  |

**Table S2.** Weighted univariate analysis of depression

|  | N | DEPRESSION |
| --- | --- | --- |
| Age (years) 分组 |  |  |
| <60 | 8940 (66.97%) | 1.0 |
| >=60 | 4410 (33.03%) | 0.86 (0.82, 0.91) <0.0001 |
| Sex |  |  |
| Male | 7058 (52.87%) | 1.0 |
| Femal | 6292 (47.13%) | 1.76 (1.69, 1.84) <0.0001 |
| Race |  |  |
| Mexican American | 2046 (15.33%) | 1.0 |
| Other Hispanic | 1292 (9.68%) | 1.60 (1.43, 1.78) <0.0001 |
| Non-Hispanic White | 6100 (45.69%) | 1.09 (1.00, 1.18) 0.0388 |
| Non-Hispanic Black | 2675 (20.04%) | 1.63 (1.48, 1.79) <0.0001 |
| Other Race | 1237 (9.27%) | 1.22 (1.09, 1.37) 0.0004 |
| Education level |  |  |
| Less than 9th grade | 1172 (8.79%) | 1.0 |
| 9-11th grade | 1896 (14.21%) | 0.75 (0.69, 0.82) <0.0001 |
| High school graduate/GED or equivalent | 3092 (23.18%) | 0.56 (0.51, 0.60) <0.0001 |
| Some college or AA degree | 3996 (29.96%) | 0.54 (0.50, 0.59) <0.0001 |
| College graduate or above | 3184 (23.87%) | 0.20 (0.18, 0.22) <0.0001 |
| Household income |  |  |
| 0–130% FPL | 3635 (29.56%) | 1.0 |
| >130–350% FPL | 4696 (38.19%) | 0.40 (0.38, 0.42) <0.0001 |
| >350% FPL | 3966 (32.25%) | 0.22 (0.21, 0.23) <0.0001 |
| BMI status |  |  |
| Normal or low weight | 3830 (28.93%) | 1.0 |
| Overweight | 4441 (33.54%) | 0.74 (0.70, 0.79) <0.0001 |
| Obese | 4968 (37.53%) | 1.41 (1.34, 1.48) <0.0001 |
| Smoking status |  |  |
| Never | 6581 (49.30%) | 1.0 |
| Former | 3738 (28.00%) | 1.37 (1.30, 1.45) <0.0001 |
| Current | 3031 (22.70%) | 3.30 (3.15, 3.47) <0.0001 |
| Drinking status |  |  |
| None | 2930 (21.95%) | 1.0 |
| Light | 6012 (45.03%) | 0.68 (0.64, 0.72) <0.0001 |
| Moderate | 2797 (20.95%) | 0.48 (0.45, 0.51) <0.0001 |
| Heavy | 1611 (12.07%) | 0.82 (0.76, 0.88) <0.0001 |
| Triglyceride (mg/dL) | 128.72 ± 109.68 | 1.00 (1.00, 1.00) <0.0001 |
| HDL cholesterol (mg/dL) | 54.00 ± 16.42 | 0.99 (0.99, 0.99) <0.0001 |
| TyG index | 9.33 ± 0.68 | 1.35 (1.31, 1.39) <0.0001 |
| Glucose (mg/dL) | 109.35 ± 35.67 | 1.00 (1.00, 1.01) <0.0001 |
| Diabetes |  |  |
| Yes | 1687 (12.65%) | 1.0 |
| no | 11332 (84.95%) | 0.54 (0.50, 0.57) <0.0001 |
| Borderline | 321 (2.41%) | 0.86 (0.75, 0.98) 0.0278 |
| coronary heart disease |  |  |
| Yes | 558 (4.20%) | 1.0 |
| No | 12743 (95.80%) | 0.62 (0.56, 0.68) <0.0001 |
| liver condition |  |  |
| Yes | 588 (4.41%) | 1.0 |
| No | 12731 (95.59%) | 0.37 (0.35, 0.40) <0.0001 |
| congestive heart failure |  |  |
| Yes | 438 (3.29%) | 1.0 |
| No | 12884 (96.71%) | 0.35 (0.32, 0.38) <0.0001 |
| cancer or malignancy |  |  |
| Yes | 1259 (9.44%) | 1.0 |
| No | 12083 (90.56%) | 0.94 (0.88, 1.00) 0.0618 |

**Table S3.** Weighted relationship between Triglyceride, glucose and depression.

| Exposure | Non-adjusted | Adjust I | Adjust II |
| --- | --- | --- | --- |
| Glucose (mg/dL) group |  |  |  |
| 21 - 92 | 1.0 | 1.0 | 1.0 |
| 93 - 100 | 0.94 (0.85, 1.03) 0.1686 | 1.05 (0.96, 1.16) 0.3023 | 1.15 (1.04, 1.27) 0.0082 |
| 101 - 110 | 1.31 (1.19, 1.43) <0.0001 | 1.61 (1.46, 1.77) <0.0001 | 1.58 (1.43, 1.76) <0.0001 |
| 111 - 584 | 1.39 (1.26, 1.52) <0.0001 | 1.75 (1.58, 1.93) <0.0001 | 1.28 (1.13, 1.45) <0.0001 |
| Glucose (mg/dL) group trend | <0.0001 | <0.0001 | <0.0001 |
| Triglyceride (mg/dL) group |  |  |  |
| 10 - 70 | 1.0 | 1.0 | 1.0 |
| 71 - 103 | 1.14 (1.03, 1.25) 0.0089 | 1.20 (1.09, 1.33) 0.0001 | 1.07 (0.96, 1.19) 0.2161 |
| 104 - 152 | 1.20 (1.09, 1.32) 0.0002 | 1.33 (1.20, 1.46) <0.0001 | 1.04 (0.94, 1.16) 0.4521 |
| 153 - 2742 | 1.72 (1.58, 1.88) <0.0001 | 2.01 (1.84, 2.21) <0.0001 | 1.45 (1.30, 1.62) <0.0001 |
| Triglyceride (mg/dL) group trend | <0.0001 | <0.0001 | <0.0001 |
| Non-adjusted model adjusts for: None  Adjust I model adjust for: Age (years); Sex; Race  Adjust II model adjust for: Age (years); Sex; Race; Education level; Smoking status; Household income; BMI status; Diabetes; coronary heart disease; congestive heart failure; liver condition; cancer or malignancy; HDL cholesterol (mg/dL) | | | |

**Table S4. Baseline characteristics between Exclude and Include individual**

|  | Exclude | Include | P-value |
| --- | --- | --- | --- |
| N | 27146 | 13350 |  |
| Age (years) group |  |  | <0.001 |
| <60 | 18719 (69.0%) | 8940 (67.0%) |  |
| >=60 | 8427 (31.0%) | 4410 (33.0%) |  |
| Sex |  |  | <0.001 |
| Male | 12575 (46.3%) | 7058 (52.9%) |  |
| Femal | 14571 (53.7%) | 6292 (47.1%) |  |
| Race |  |  | <0.001 |
| Mexican American | 4486 (16.5%) | 2046 (15.3%) |  |
| Other Hispanic | 2566 (9.5%) | 1292 (9.7%) |  |
| Non-Hispanic White | 10432 (38.4%) | 6100 (45.7%) |  |
| Non-Hispanic Black | 6287 (23.2%) | 2675 (20.0%) |  |
| Other Race | 3375 (12.4%) | 1237 (9.3%) |  |
| Education level |  |  | <0.001 |
| Less than 9th grade | 2998 (12.1%) | 1172 (8.8%) |  |
| 9-11th grade | 3577 (14.4%) | 1896 (14.2%) |  |
| High school graduate/GED or equivalent | 5658 (22.8%) | 3092 (23.2%) |  |
| Some college or AA degree | 7153 (28.8%) | 3996 (30.0%) |  |
| College graduate or above | 5408 (21.8%) | 3184 (23.9%) |  |
| Household income |  |  | <0.001 |
| 0–130% FPL | 8528 (34.9%) | 3635 (29.6%) |  |
| >130–350% FPL | 9086 (37.1%) | 4696 (38.2%) |  |
| >350% FPL | 6854 (28.0%) | 3966 (32.3%) |  |
| BMI status |  |  | <0.001 |
| Normal or low weight | 8457 (31.8%) | 3830 (28.9%) |  |
| Overweight | 8494 (31.9%) | 4441 (33.5%) |  |
| Obese | 9685 (36.4%) | 4968 (37.5%) |  |
| Smoking status |  |  | <0.001 |
| Never | 15382 (59.9%) | 6581 (49.3%) |  |
| Former | 5419 (21.1%) | 3738 (28.0%) |  |
| Current | 4877 (19.0%) | 3031 (22.7%) |  |
| Drinking status |  |  | 0.584 |
| None | 3722 (22.1%) | 2930 (21.9%) |  |
| Light | 7686 (45.6%) | 6012 (45.0%) |  |
| Moderate | 3485 (20.7%) | 2797 (21.0%) |  |
| Heavy | 1964 (11.7%) | 1611 (12.1%) |  |
| Triglyceride (mg/dL) | 117.1 ± 106.6 | 128.7 ± 109.7 | <0.001 |
| HDL cholesterol (mg/dL) | 52.6 ± 15.9 | 54.0 ± 16.4 | <0.001 |
| Glucose (mg/dL) | 108.1 ± 37.8 | 109.4 ± 35.7 | 0.034 |
| Diabetes |  |  | 0.017 |
| Yes | 3280 (12.1%) | 1687 (12.6%) |  |
| no | 23288 (85.9%) | 11332 (84.9%) |  |
| Borderline | 558 (2.1%) | 321 (2.4%) |  |
| coronary heart disease |  |  | 0.443 |
| Yes | 997 (4.0%) | 558 (4.2%) |  |
| No | 23732 (96.0%) | 12743 (95.8%) |  |
| liver condition |  |  | 0.005 |
| Yes | 946 (3.8%) | 588 (4.4%) |  |
| No | 23845 (96.2%) | 12731 (95.6%) |  |
| congestive heart failure |  |  | 0.573 |
| Yes | 841 (3.4%) | 438 (3.3%) |  |
| No | 23917 (96.6%) | 12884 (96.7%) |  |
| cancer or malignancy |  |  | 0.781 |
| Yes | 2363 (9.5%) | 1259 (9.4%) |  |
| No | 22448 (90.5%) | 12083 (90.6%) |  |
| Depression |  |  | 0.074 |
| No | 20967 (90.7%) | 12181 (91.2%) |  |
| Yes | 2154 (9.3%) | 1169 (8.8%) |  |

**Table S5. Missing of variables**

| Variables | Not Missing | Missing |
| --- | --- | --- |
| Depression | 36471 | 4025 |
| TyG index | 18002 | 22494 |
| Age (years) | 40496 | 0 |
| Sex | 40496 | 0 |
| Education level | 38134 | 2362 |
| Race | 40496 | 0 |
| SMOKING.STATUS | 39082 | 1468 |
| Household income | 36765 | 3731 |
| Drinking status | 30207 | 10289 |
| BMI status | 39875 | 621 |
| Diabetes | 40466 | 30 |
| coronary heart disease | 38030 | 2466 |
| liver condition | 38110 | 2386 |
| congestive heart failure | 38080 | 2416 |
| cancer or malignancy | 38153 | 2343 |
| Glucose (mg/dL) | 18544 | 21952 |
| Triglyceride (mg/dL) | 18028 | 22468 |
| HDL cholesterol (mg/dL) | 37952 | 2544 |
